# Fibroblasts as Playmakers of Cancer Progression: Current Knowledge and Future Perspectives

**DOI:** 10.3390/cancers15235538

**Published:** 2023-11-22

**Authors:** Kenichiro Ishii

**Affiliations:** 1 Department of Nursing, Nagoya University of Arts and Sciences, 4-1-1 Sannomaru, Naka-ku, Nagoya 460-0001, Japan; kenishii@nuas.ac.jp; Tel.: +81-52-954-1222; 2 Department of Oncologic Pathology, Mie University Graduate School of Medicine, 2-174 Edobashi, Tsu 514-8507, Japan

This series of six articles (four original articles and two reviews) is presented by international leaders in stromal biology in the tumor microenvironment. This original series of articles details the important role of fibroblasts in cancer progression, focusing on current knowledge and future perspectives.

In homeostatic epithelial–stromal interactions in adult tissues, stromal paracrine signals (morphogens) act to maintain the functional differentiation and growth/quiescence of epithelial cells [1]. Once cancer cells begin proliferating in the epithelial compartment, deregulation of homeostatic interactions occurs, leading to structural alterations in the stroma called stromal remodeling [1]. In contrast, stroma-induced malignant transformation of epithelial cells has been experimentally reported in prostate cancer development, suggesting that the stromal structure plays a critical role in cancer development and primary cancer cell progression [2].

In the stroma of various solid tumors, invading cancer cells interact in complex ways with each other or with the surrounding microenvironment, generating the reactive stroma [3]. The reactive stroma is composed mainly of cancer-promoting fibroblasts, known as cancer-associated fibroblasts (CAFs). Within a focal lesion, CAFs heterotypically communicate with cancer cells not only by direct cell–cell contact via cell adhesion molecules, but also by indirect cell–cell communication via mitogenic soluble factors, including growth factors, cytokines, extracellular matrix molecules, and miRNAs [1]. Interestingly, recent studies have unveiled that the reactive stroma of pancreatic cancer contains multiple functionally diverse populations of fibroblasts that positively or negatively regulate cancer progression (i.e., cancer-promoting or cancer-restraining) [4]. 

The collection includes two insightful reviews discussing stromal biology in the tumor microenvironment. In one of the reviews, Wieder discusses how cancer cells influence the character of the most abundant cells in the stroma, i.e., fibroblasts, and in turn how the altered fibroblasts enhance cancer’s aggressiveness [5]. This review also outlines efforts to use these altered fibroblasts as new targets for cancer treatment. In the other review, Glabman et al. summarize the current research, highlight major challenges, and discuss future opportunities to improve our knowledge of CAF biology and therapeutic targeting [6]. This review summarizes the recent and major advances in CAF-targeting therapies, including DNA-based vaccines, anti-CAF CAR-T cells, and modification and reprogramming of CAF functions. The challenges of developing effective anti-CAF treatments include CAF heterogeneity and plasticity, a lack of specific target markers for CAFs, limitations in animal models recapitulating the human tumor microenvironment, and undesirable off-target and systemic side effects.

Several of the articles highlight the cancer-promoting roles of fibroblasts. Millet et al. proposed a 3D self-assembly engineered bladder model using CAFs, in which stromal cells produced their extracellular matrix [7]. They proceeded to assess how their model recapitulates the biological and mechanical features found in tumors. In an attempt to identify fibroblast-derived molecular targets for cancer progression, Huang et al. showed that CAFs overexpress chemokine ligand 11 (CCL11), which is associated with tumor migration and invasion, increased expression of cancer stem cell properties, and induction of the epithelial-to-mesenchymal transition [8]. Their results suggest that targeting CCL11/CCR3 signaling is a potential therapeutic strategy for patients with aggressive head and neck cancer. In addition, Schulze et al. demonstrated that NT5DC2 expression tends to be associated with more abundant SMA-positive CAFs, while p53 expression is inversely associated with the presence of CD34-positive CAFs [9]. For developing molecular imaging and therapies, Kosmala et al. quantitatively determined the normal organ biodistribution of [68Ga]Ga-FAPI-04 according to the tumor extent and assessed its relationship with the FAPI-avid tumor burden [10].

In the tumor microenvironment, heterotypic interactions between cancer cells and fibroblasts are likely very important for determining cancer cell behavior, but we still know very little about the biological role of fibroblasts in the reactive stroma [11,12]. In this Special Issue, we focused on heterotypic interactions between cancer cells and fibroblasts to initiate the design of reactive stroma-targeted therapies for the treatment of primary solid tumors. Altering the malignant phenotype of cancer-promoting fibroblasts, for example, via differentiation therapy targeting the reactive stroma may abrogate primary cancer cell progression. We hope that this Special Issue attracts the attention of readers with expertise and interest in fibroblasts as playmakers of cancer progression.
